# Patterned Fibers Embedded Microfluidic Chips Based on PLA and PDMS for Ag Nanoparticle Safety Testing

**DOI:** 10.3390/polym8110402

**Published:** 2016-11-16

**Authors:** Yaowen Liu, Shuyao Wang, Yihao Wang

**Affiliations:** 1 College of Food Science, Sichuan Agricultural University, Yaan 625014, China; shuyaow@163.com (S.W.); cdyihaow@163.com (Y.W.); 2 School of Materials Science and Engineering, Southwest Jiaotong University, Chengdu 610031, China

**Keywords:** patterned fibers, microfluidic chips, hepatocyte spheroid, nano-toxicity

## Abstract

A new method to integrate poly-dl-lactide (PLA) patterned electrospun fibers with a polydimethylsiloxane (PDMS) microfluidic chip was successfully developed via lithography. Hepatocyte behavior under static and dynamic conditions was investigated. Immunohistochemical analyses indicated good hepatocyte survival under the dynamic culture system with effective hepatocyte spheroid formation in the patterned microfluidic chip vs. static culture conditions and tissue culture plate (TCP). In particular, hepatocytes seeded in this microfluidic chip under a flow rate of 10 μL/min could re-establish hepatocyte polarity to support biliary excretion and were able to maintain high levels of albumin and urea secretion over 15 days. Furthermore, the optimized system could produce sensitive and consistent responses to nano-Ag-induced hepatotoxicity during culture. Thus, this microfluidic chip device provides a new means of fabricating complex liver tissue-engineered scaffolds, and may be of considerable utility in the toxicity screening of nanoparticles.

## 1. Introduction

In recent years, nanotechnology has experienced dramatic developments leading to its wide application in areas such as solar energy, cosmetics, food production, and drug delivery [1]. However, the potential toxicity of nanoparticles remains a concern; they readily enter the body and relocate within metabolically active organs, with the liver representing the main organ for nanoparticle accumulation and biotransformation. Although many in vivo models have been developed to evaluate the potential toxicity of nanoparticles, improved methods are needed to decrease the numbers of animals required for testing along with the experimental expenses, and to improve the accuracy, comparability, and reproducibility of experimental results. However, previous studies have shown that many in vitro toxicity testing methods fail to identify the hazards of nanoparticles, as primary hepatocytes rapidly lose their morphology and specific functions—such as the activity of phase I and phase II enzymes and the production of plasma proteins—under traditional incubation methods [2]. In contrast, it has been demonstrated that hepatocytes cultured as spheroids are able to promote cell–cell communication and gap junctions, form bile canaliculi, and express specific transporters. Thus, many methods have been used to create spatial variations and topologies to mediate cell aggregates; however, these have exhibited difficulty in mimicking the exact microenvironment of hepatocytes, combating poor oxygen and nutrient diffusion [3,4].

To support long-term maintenance of hepatocytes, researchers have used perfusion culture systems to supply transport proteins and large molecules to cultured hepatocytes [5]. However, these systems all showed inherent limitations such as high shear, lack of physiologically relevance, requirement for high cell numbers, and low viability of hepatocytes [6]. To overcome these restrictions, many miniaturization perfusion systems were developed to sufficiently mimic the in vivo situation, which is an important consideration for liver tissue engineering and pharmacological drug screening. In addition, miniaturization perfusion systems offer a very well-suited microenvironment for cultured cells, allowing the control of features such as culture media, velocity of cell culture fluid, and liquid concentration [7].

In native liver tissues, hepatocytes are arranged into a single cordlike structure and separated by adjacent sinusoids [8]. Many researchers have attempted to mimic the microenvironment of hepatocytes, including oxygen content, nutrient delivery, metabolite removal, and shear stress. Sudo et al. cultured primary hepatocytes and rat/human microvascular endothelial cells (MVECs) on each sidewall of a collagen-gel scaffold between two microfluidic channels, demonstrating that hepatocytes and MVECs could exchange secreted proteins by diffusional transport under flow conditions [9]. Zhang et al. reported that primary hepatocytes on a galactosylated microporous collagen-gel membrane could preserve the original morphology of hepatocytes and maintained high levels of urea production and cytochrome P450 activity for 14 days [10]. Lee et al. also developed a 3D liver-on-a-chip platform to investigate the interaction of hepatocytes and hepatic stellate cells. Notably, this liver chip was supplied with a continuous flow of medium through osmotic pumping, which played an important role in the formation of tight hepatocyte spheroids and thereby improved liver-specific function over a long duration [11]. However, these systems for hepatocyte cultivation comprise 2D monolayer cultures, and cells embedded in such substrates are not reflective of 3D cultivation conditions [12]. Additionally, they provide only limited long-term hepatocyte cultures for investigating protective drug effects and the underlying mechanisms of drug toxicity [13]. Although several 3D scaffolds have been developed to facilitate the establishment of microfluidic channels to maintain hepatocyte function, remaining obstacles include the inability of many scaffolds to offer a defined stiffness for hepatocytes, the lack permeability for the transport of large macromolecules, and hampered cell–cell interactions [14]. 

Electrospun fibers have received increasing attention over last several decades as a component of cell culture scaffolding; in particular, polymer fibers have offered several advantages in mimicking the size scale of fibrous extracellular matrix (ECM) and providing a very high fraction of surface available for cell interaction. As microscale interconnected pores are essential to transport oxygen and nutrient supplies for cell growth and are beneficial for the adhesion and viability of primary hepatocytes [3], electrospun fibers have emerged as promising candidates and useful substrates in the field of hepatic tissue engineering. Several researchers have shown that the incorporation of electrospun fibrous mats into microfluidic chips could be widely used in cell culture. For example, Lee et al. developed a polydimethylsiloxane-based microfluidic chip and evaluated the feasibility of human bone marrow-derived mesenchymal stem cell culture under perfusion conditions [15]. Patric et al. utilized lithography methods to integrate patterned electrospun fiber pads with microfluidic networks, yielding good attachment and growth of fibroblasts [16]. In addition, microfluidic networks have also been shown to efficiently generate spatially and temporally defined liquid microenvironments. Furthermore, electrospun fiber-based microfluidic networks exhibit the potential for wide use in bioassays as well. Because electrospun fibrous mats possess high specific surface area and macro- and mesoporous structures, they exhibit high levels of protein absorbance. For example, Liu et al. assembled electrospun nanofibrous polyvinylidene fluoride (PVDF) membranes into a polydimethylsiloxane (PDMS) chip and found that the composite microfluidic system increased the levels of protein adsorption and improved the sensitivity in immunoassays [17].

Despite such advances, the integration of microfluidic chips with fibrous mats systems has not been reported for use in hepatocyte culture. Poly-dl-lactide (PLA) is a synthetic polyester, its chemical composition provides a hydrophobic nature and linear structure which contributes to an excellent spinability process during electrospinning. Furthermore, it is biocompatible, biodegradable, and nontoxic. In this study, we developed and evaluated a method to integrate PLA micropatterned fibers with microfluidic networks. Primary hepatocytes were cultured in micropatterned fibrous mats embedded in 3D microfluidic chips and the behaviors of the hepatocytes were investigated to measure the effects of the interplay of topography, substrate surface properties, and soluble microenvironments. The underlying hypothesis of this work is that the optimized perfused culture system might offer multifunctional benefits and provide a biomimetic hepatocyte culture environment, thus enabling long-term maintenance of hepatocytes with high degrees of liver-specific characteristics. Here, the utility of the optimized perfused cultures in ascertaining the toxic effects of silver nanoparticles (nano-silver; Ag NPs) was tested in comparison with the determinations established by conventional culture-based cytotoxicity. The system as described in this study thereby represents an important platform for studying or controlling phenotype expression in tissue engineering and for toxicity screening of nanoparticles.

## 2. Materials and Methods

### 2.1. Materials

Poly-dl-lactide (PLA, *M*_W_ = 180 kDa) was synthesized in our laboratory. PDMS (Sylgard 184 Silicone kit) was purchased from Dow Corning (Midland, MI, USA). Rhodamine B and collagenase IV were procured from Sigma Aldrich (St. Louis, MO, USA). Calcein-AM, propidium iodide, and fluorescein diacetate (FDA) were purchased from Molecular Probes (Carlsbad, CA, USA). Anti-goat IgG–FITC (fluorescein isothiocyanate) was purchased from Biosynthesis Biotechnology Co., Ltd. (Beijing, China). Ag NPs (particle size 20–40 nm) were purchased from Nanonasb-Pars Company (Tehran, Iran). All chemical reagents used were of analytical grade and obtained from Chengdu Kelong Reagent Co. (Chengdu, China) unless otherwise indicated.

### 2.2. Fabrication of Microfluidic Chips

Microfluidic chips were fabricated by standard soft lithography. Briefly, the microchannel structure was designed using L-edit (Tanner EDA, Wilsonville, OR, USA) and produced with a chrome photomask using an E-beam mask lithography system (Mark 40, CHA Industries, Fremont, CA, USA). The mask was utilized to photo pattern 400 μm thick spin coated SU-8 (SU-8 2050, MicroChem Co., Newton, MA, USA) onto silicon wafers (Tianjin Institute of Semiconductors, Tianjin, China). The fabricated mold was then silanized with 1H, 1H, 2H, 2H-perfluorooctyltrichlorosilane (78560-45-9, AlfaAesar, Ward Hill, MA, USA) in a desiccator for more than 30 min at room temperature to prevent undesired bonding of PDMS to the mold. PDMS prepolymer with 1:10 (*v*/*v*) curing agent to base ratio was poured on the mold and cured for 2 h at 95 °C to obtain the final microchannel structure.

A biopsy punch with an inner diameter of 1.0 mm was used to punch holes through the PDMS for the inlet and outlet ports. The microchannels of the microfluidic chip were 200 μm wide and approximately 3 mm long. The height of the channels was 400 μm throughout the whole network. The PDMS device was then cured in a 60 °C oven for more than 2 h to promote the bonding and ensure full curing of the PDMS to enhance cell compatibility.

### 2.3. Fabrication of Patterned Fibers

Previous studies have shown that patterned fibers with a width of 200 μm and a maximum thickness of approximately 100 μm could provide a platform to maintain the viabilities and functions of hepatocytes [18]. To form fluid channels across the patterned fibers and reconstruct the perfused engineered liver, microchannels sized 200 µm wide and 400 µm high were chosen and designed. The patterned collector was constructed on a glass template patterned with an electrically conductive circuit as previously described [4]. In brief, an insulating glass substrate was coated with a silver layer by direct-current (DC) sputtering (Sunicoat 594L, Sunic System Ltd., Sokcho, Korea), coated with a second layer of photoresist (MicroChem Inc., Newton, MA, USA), then covered with the photomask containing parallel strips of 200 μm in width, and exposed using a lithography machine (Suss Mircotec MA6, SUSS MicroTec, Garching, Germany). As the exposed regions became soluble, the glass substrate was rinsed to remove the photoresist followed by etching away silver in the exposed area. After removing the rest of the photoresist, the glass patterned collector was obtained as a collector for the electrospinning process. PLA was dissolved in chloroform at 15 wt %. The polymer solution was added into a 2 mL syringe attached with a metal capillary shaped for clinical use. A syringe pump (Zhejiang University Medical Instrument Company, Hangzhou, China) was used to feed the polymer solution to maintain a steady flow at 1 mL/h from the capillary outlet. The distance between the capillary tip and patterned collector was set to about 15 cm and the electrospinning voltage was controlled within 20 kV using a high-voltage statitron (Tianjin High Voltage Power Supply Company, Tianjin, China) to obtain the patterned PLA fibers.

### 2.4. Construction of Perfusion Systems

To ensure that the patterned fibrous mats were placed in the center of the probing channel, the PDMS channel was aligned manually using a stereomicroscope. The bottom layer was aligned and irreversibly bonded with the top layer using 3M Scotch Tape [17], and short Teflon tubing (Genetec AB, Västra Frölunda, Sweden) was glued (Elastosil A07, Wacker Silicones AG, München, Germany) on the inlet and outlet ports. They were later used to connect the microfluidic chip to the pump. The pump setup used was designed to control the flow rate for each inlet and was assembled in our lab. After the assembly of the chip, rhodamine B fluorescent dye was perfused by the pump system into the microchannel to determine whether the system exhibited problems of liquid leakage. We also used the pump system to place a drop of solution on one inlet of the channels and to remove solutions from these microchannels by applying vacuum at the outlets.

### 2.5. Cell Culture and Microscopic Investigations

Prior to use in cell culture, the bonded microfluidic channel was sterilized with 75% ethanol for 10 min and washed extensively with Dulbecco’s Modified Eagle Medium (DMEM). Primary hepatocytes were isolated from the livers of adult SD rats using a two-step collagenase perfusion procedure [19]. Male SD rats weighting 120–150 g were purchased from Sichuan Dashuo Biotech Inc. (Chengdu, China) and all animal protocols were approved by the University Animal Care and Use Committee. Only hepatocytes with viability of >90%, as determined by the trypan blue exclusion assay, were used. A primary hepatocyte suspension (500 μL of 1 × 10^3^ cells/μL) in medium (DMEM with 10% (*v*/*v*) fetal bovine serum (FBS)) was loaded into the microchannel from the inlet and the entire process was monitored under a microscope. The cells were allowed to attach for 10 h without any flow; subsequently, fresh medium from the inlets was re-established at a varied flow rate for each channel. The perfusion system was then placed in an incubator with 5% CO_2_ at 37 °C for 15 days. For analysis, at each time point, the microchannel was washed with phosphate-buffered saline (PBS) and the cells were fixed with 4% formaldehyde for 2 h at 4 °C under static conditions. The viability of hepatocytes cultured within the device was analyzed using a live/dead assay as described previously. Briefly, the microfluidic system was incubated with 50 mM calcein-AM and 25 mg/mL propidium iodide for 30 min at 37 °C in the dark, then the sample was washed extensively with PBS and observed under a confocal laser scanning microscope (CLSM, Olympus FV1000S, Tokyo, Japan).

To investigate the feasibility of assembly of microfluidic chips and patterned fibers, the system was incubated with 1 mg/mL rhodamine B solution overnight. After washing with distilled water, the microfluidic chips with embedded patterned fibers were observed by CLSM under the excitation and emission wavelengths of 550 and 620 nm for rhodamine B. The patterning features of the electrospun fibrous mats and microfluidic chips were observed using an optical microscope (Nikon Eclipse TS100, Tokyo, Japan). The morphologies of the electrospun fibers were investigated using a scanning electron microscope (SEM, FEI Quanta 200, Eindhoven, The Netherlands) equipped with a field-emission gun and Robinson detector after 2 min of gold coating to minimize the charging effect. The spheroid sizes were evaluated after processing the CLSM images by Image-Pro 6.0 (Media Cybernetics Inc., Bethesda, MD, USA) [3]. Briefly, CLSM images were taken by z-stack scanning with a step size of 5 μm. The images were stacked and reconstructed by Image-Pro 6.0, the desired spheroid for counting was selected, and counting was carried out by clicking on the fluorescein localization in spheroid in the image. The total area covered by calcein-AM-containing cells were displayed on the image. 

### 2.6. Assessment of Hepatocyte Function

Albumin and urea secretion were analyzed by measuring the concentration of albumin and urea in the media. After culturing for 3, 7, 11, and 15 days, the albumin secretion was measured by enzyme-linked immunosorbent assay (ELISA) using a commercial rat albumin quantitation kit (Nanjing Jiancheng Bioengineering Institute, Nanjing, China). Urea levels were measured with commercially available kits (Nanjing Jiancheng Bioengineering Institute) based on its specific reaction with diacetylmonoxime. Absorbance was measured with a Quant microplate spectrophotometer (Elx-800, BioTek Instrument Inc., Winooski, VT, USA) and standard curves were generated using purified rat albumin or urea dissolved in culture media. All functional data were normalized to 10^6^ cells. The biliary excretion of hepatocytes was determined by FDA staining following procedures described previously [3]. Briefly, hepatocytes cultured within the device were incubated in culture media containing 3 mg/mL FDA at 37 °C for 1 h. After extensive washes with PBS, samples were observed by CLSM under the excitation and emission wavelengths of 488 and 533 nm, respectively. These images were processed by Image-Pro 6.0 to quantify the fluorescein localization in the intercellular sacs between hepatocytes. The biliary excretion of an FDA-staining image was indicated by the ratio of the area of excreted fluorescein in the intracellular sacs to the total area covered by FDA-containing cells; more than five original images were randomly chosen for each sample.

### 2.7. Nanoparticle-Induced Hepatotoxicity Testing

In order to obtain a homogeneous dispersion of Ag NPs (no color change and no precipitation), the Ag NPs were stably diluted with deionized water using physical mixing and sonication several times, and then sterilized by passing the solution through a 0.22-micron microfilter. Finally, Ag NPs (120 μg/mL) were prepared in DMEM without serum [20]. After the hepatocytes were exposed to nanoparticles for 24 h, the various toxicity endpoints were evaluated in control and nanoparticle-exposed cells.

The membrane damage of the Ag NP-exposed cells was assessed by measuring the activity of lactate dehydrogenase (LDH) in the cells and media using a commercial diagnostic kit (Nanjing Jiancheng Bioengineering Institute, Nanjing, China) as previously described [4]. Briefly, after culturing for 7 and 15 days, the media were collected and centrifuged. The activity of the LDH released from the cytosol of damaged cells was assessed using the LDH kit and the optical density at 450 nm was measured using an Elx-800 spectrophotometer (BioTek Instrument Inc., Winooski, VT, USA). The total LDH release of cells was determined after lysis in 0.8% polyoxyethylene octyl phenyl ether (Triton^TM^ X-100) at 37 °C for 45 min. The LDH leakage after Ag NP treatment for 24 h was normalized to the value without Ag NP treatment.

### 2.8. Statistical Methods

All the data presented are expressed as the mean ± standard deviation (SD), and analysis of variance (ANOVA) were used for statistical evaluation of the data. A probability value (*p*) of less than 0.05 was considered to be statistically significant. 

## 3. Results

### 3.1. Assembly of PDMS Chip with PLA Patterned Fibers

**Figure 1:**
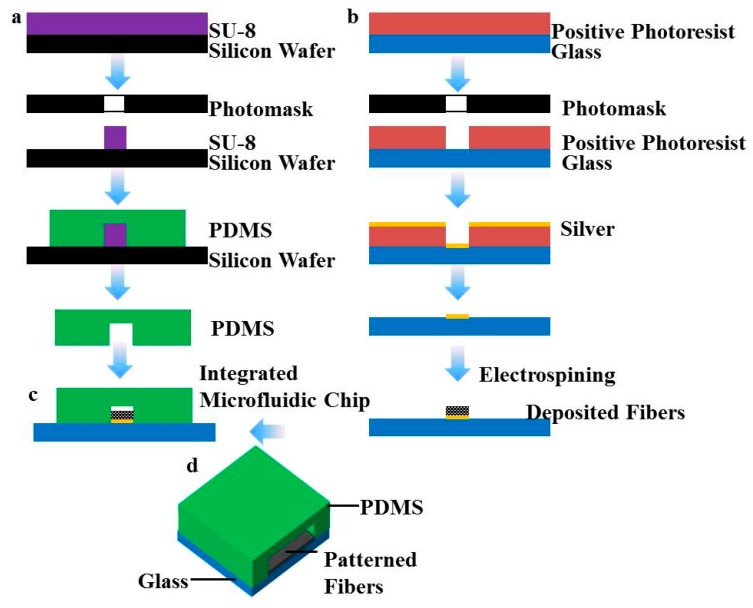
Schematic illustration of the microfabricated (**a**) patterned fibers and (**b**) microfluidic chips; (**c**) microfluidic chip integrated with patterned fibers; (**d**) 3D schematic illustration of a patterned fiber-embedded microfluidic chip.

Figure 1 shows the schematic illustration of the fabrication process of a patterned fiber-embedded microfluidic chip. The device was constructed using two layers: the bottom layer with patterned fibers and a channel in the top layer covering the patterned fiber chambers. Firstly, the top layer was fabricated from PDMS by standard soft lithography methods. Briefly, microfluidic networks were drawn by Tanner L-Edit software and printed with high resolution on a photomask by an E-beam mask lithography system. The convex rectangular master mold was replicated by using SU-8 followed with UV exposure and a PDMS layer with a rectangular channel structure was created by the separation of SU-8 from the PDMS base mold (Figure 1a). The bottom layer of patterned PLA fibers was obtained using a patterned collector as described previously, with some modifications [3]. Briefly, an insulating glass substrate was coated with a layer of positive photoresist, then covered with the photomask and exposed using a lithography machine. A silver layer was then deposited on the glass substrate by DC sputtering. After removing the rest of the photoresist, the glass substrate, representing a micropatterned silver circuit, served as a collector for the electrospinning process (Figure 1b). Finally, the top and bottom layer chambers were aligned and bonded via 3M Scotch Tape [17], which was necessary for creating the coaxial-flow channels without clogging (Figure 1c). The 3D schematic illustration of the patterned fiber-embedded microfluidic chip is shown in Figure 1d.

### 3.2. Optimization of Patterned Fiber-Embedded Microfluidic Chips

Previous studies have shown that patterned fibers with a width of 200 μm could provide a platform to maintain the viability and function of hepatocytes [4]. To form a fluid channel across the patterned fibers and reconstruct the perfused engineered liver, microchannels sized 200 µm wide and 400 µm high were designed.

**Figure 2:**
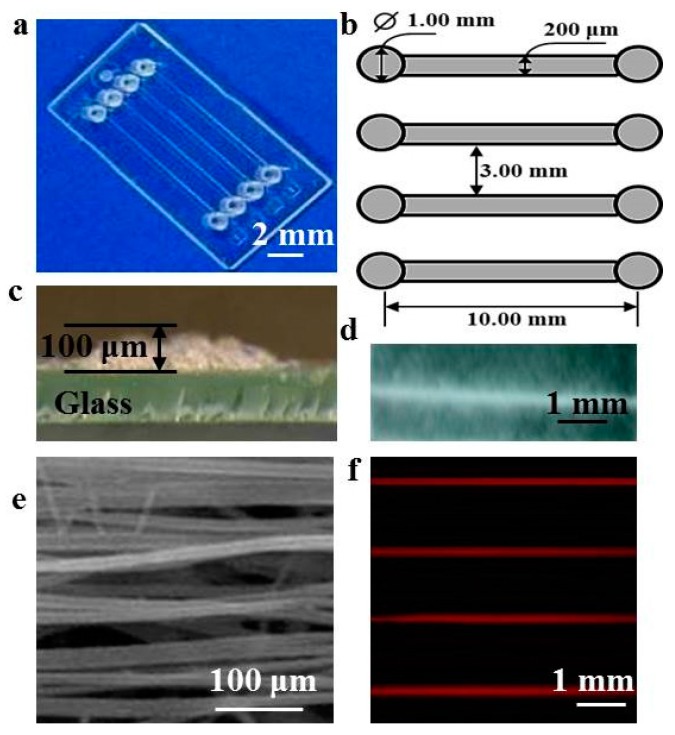
(**a**) Digital image of patterned fiber-embedded microfluidic chip; (**b**) schematic illustration of the perfusion device design; (**c**) optical image of a vertical section of patterned fibers deposited on the glass; (**d**) digital image of patterned fibers; (**e**) SEM image of patterned fibers; (**f**) fluorescence image of rhodamine B dye within the microfluidic device.

Figure 2a shows an image of the patterned fiber-embedded microfluidic chip. The device contained four rectangular microchannels 10.0 mm long and 200 μm wide; parallel microchannels were separated by a 3.0 mm gap (Figure 2b). The topography of the patterned fibrous mats was close to that of the microchannels and the thickness of the PLA fibrous mats was approximately 100 μm, which did not block the flow (Figure 2c). Figure 2d shows the image of a single rectangular shape of patterned fibers. Owing to electrostatic forces, patterned fibers showed a similar topological structure and dimension as the collector configuration. In addition, Coulombic interactions between the nanofibers and collector induced fibers to arrange along the strip, causing the fiber to be directionally arranged as shown in Figure 2e. To evaluate whether the system showed problems of liquid leakage, rhodamine B fluorescent dye was perfused from two side microchannels into the patterned fibrous mat-embedded microfluidic chip at a velocity of 10 μL/min, which yielded no evidence of rhodamine B leakage (Figure 2f). We then continued to increase the velocity of rhodamine B, and after injecting rhodamine B into the microchannel with a syringe at over 20 μL/min, the fluorescent dye exhibited leakage from one microchannel to another. Therefore, we chose velocities of 5, 10, and 20 μL/min for the subsequent experiments.

### 3.3. Hepatocyte Viability

**Figure 3:**
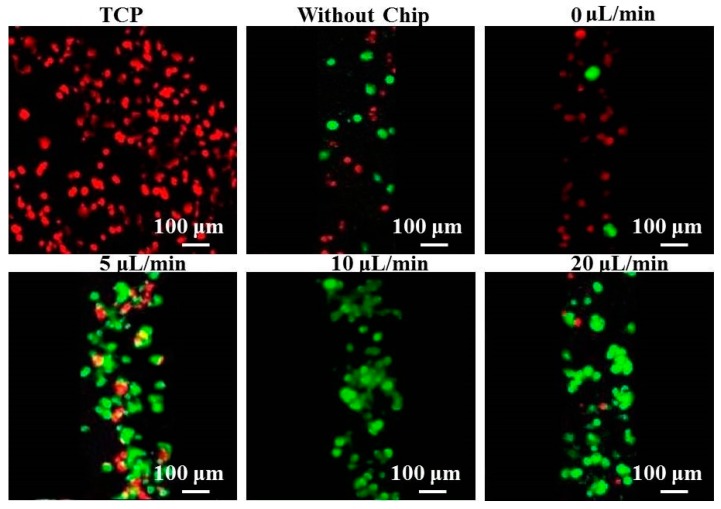
Confocal laser scanning microscope (CLSM) images of calcein-AM (**green**) and propidium iodide (**red** and **yellow**) stained hepatocytes after 3 days of culture on different substrates.

Calcein-AM and propidium iodide were used to examine hepatocyte viabilities under different flow rates (0, 5, 10, and 20 μL/min). After 3 days of culture, it was observed that all hepatocytes were dead (red and yellow signal) on tissue culture plates (TCP) (Figure 3). The number of living hepatocytes on the fibers was apparently higher than that of TCP, because hepatocytes on nanofibers generally retain preferential cell bioactivity during a 3-day culture period. However, some hepatocytes started to lose activity (yellow and yellow) under the flow rate of 0 μL/min (no flow), and we could observe abundant dead hepatocytes in the patterned fiber-embedded microfluidic chip as hepatocytes under static conditions rapidly disaggregate from the lack of nutrients. Therefore, cell viability was significantly lower in hepatocytes under static conditions than under conditions of flow, and fewer dead hepatocytes were detected under the three flow rates than static conditions after 3 days of culture. In particular, dead cells were rarely detected under the flow rate of 10 μL/min compared with other rates, demonstrating that medium-flow positively affects the viability of spheroids during the initial culture period. Death and shedding of hepatocytes occurred continuously when exposed to the flow rate of 20 μL/min. These findings are consistent with a report by Tilles et al., who showed that whereas increasing the perfusion flow rate enhances the delivery of nutrients and removal of waste, an extremely high flow rate might induce excessive wall shear stress that is detrimental to hepatocytes [21]. 

### 3.4. Time Course of Hepatocyte Size Distribution

**Figure 4:**
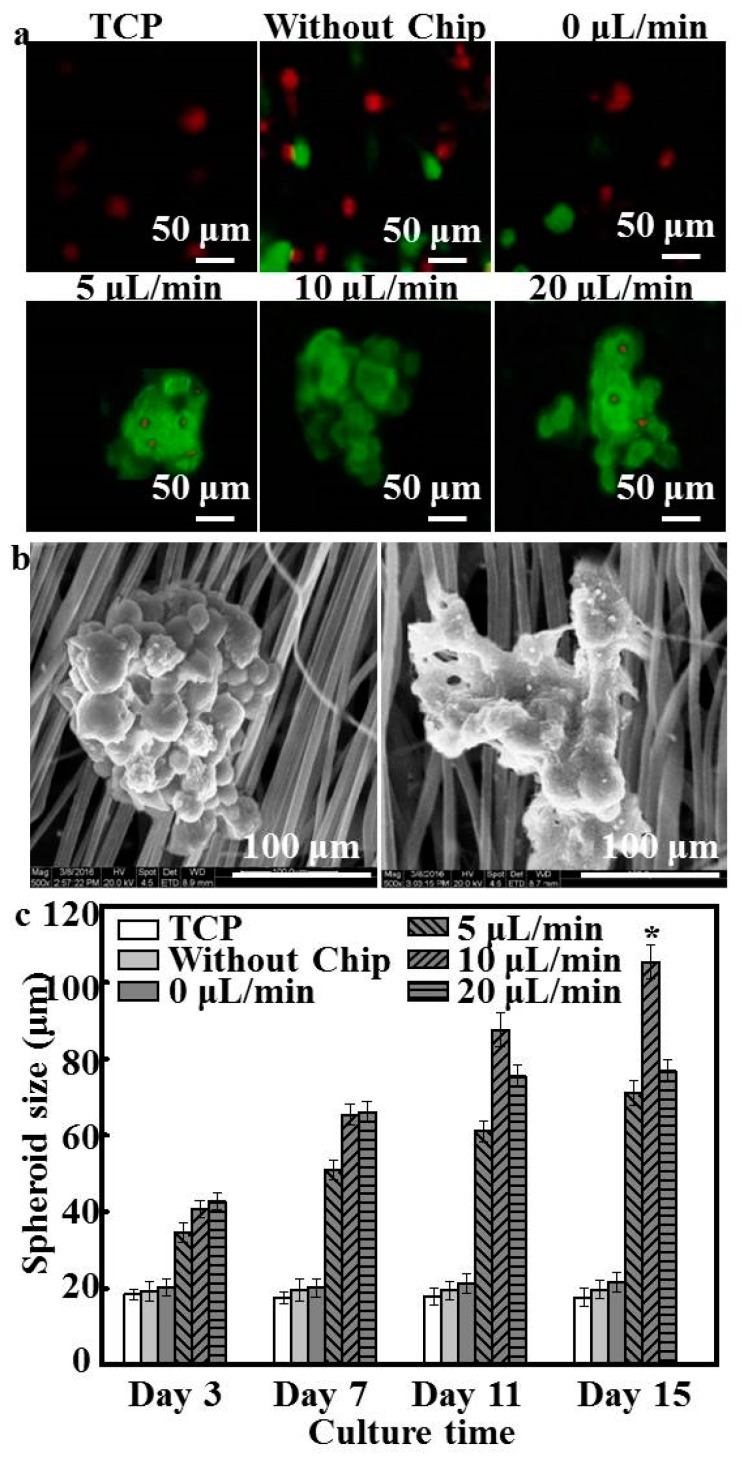
(**a**) CLSM images of spheroids formed on different substrates after 15 days in culture; (**b**) SEM images of hepatocytes under the flow rate of 10 and 20 μL/min after 15 days of incubation; (**c**) size of hepatocyte spheroids after 15 days of culture on different substrates (*n* = 5); * *p* < 0.05.

To determine the effects of flow rates on the size of spheroids, CLSM images were taken of hepatocyte spheroids formed at different culture conditions (summarized in Figure 4a). The size of hepatocytes without flow was essentially unchanged from day 7 to day 15; with death and shedding of hepatocytes occurring continuously throughout the culture period, the number of adhered hepatocytes was reduced (Figure 4a). Hepatocytes spontaneously formed spheroids after 3 days of culture in the patterned fiber-embedded microfluidic chip, wherein the hepatocyte size slightly increased with flow rates and times. In addition, fewer dead cells were detected at the flow rates of 5 and 20 μL/min compared with the others. The seeded hepatocyte aggregates perfused at the middle flow rate of 10 μL/min showed good cell bioactivity without death of inner hepatocytes during the 15-day culture period, exhibiting a compact polyhedral spheroidal morphology similar to 3D morphologies observed in vivo (Figure 4b). This indicated that the appropriate flow rate through the device exerted a marked effect on maintaining aggregation and spheroid formation. As shown in Figure 4a, spheroids at the flow rate of 20 μL/min became irregular and started to disaggregate at 15 days, whereas those at the flow rate of 10 μL/min maintained their smooth surface (reflecting tighter junctions) throughout the entire culture period, demonstrating quite substantial differences between the two rates during long-term culture (Figure 4b).

Figure 4c summarizes the sizes of the few spheroids formed after hepatocytes were cultured on static systems. Hepatocytes self-aggregated and invariably formed into spheroids when exposed to low flow rates. When hepatocytes were cultured under the flow rate of 5 μL/min, some cells formed spheroids with a size of approximately 70 μm, which was close to the results shown in Figure 4a. These findings indicated that an apparent increase in the spheroid size occurred with the increase of flow rate and flow time [21]. The majority of hepatocytes were incorporated into spheroids with an average diameter of 105.33 ± 4.54 μm after 15 days of culture at the flow rate of 10 μL/min; however, hepatocyte spheroids formed at the flow rate of 20 μL/min showed small amounts of spheroid disaggregation, significantly lower than that observed at the flow rate of 10 μL/min (*p* < 0.05). Previous studies have shown that the size of aggregates was primarily determined by the flow rate and that primary hepatocytes were particularly sensitive to the shear stress induced by the flow dynamics of the microfluidic environment [22]. In our study, the flow rate of 20 μL/min might have accelerated the detachment and the death of the cells inside the device after 11 days.

### 3.5. Evaluation of Hepatocyte Function

**Figure 5:**
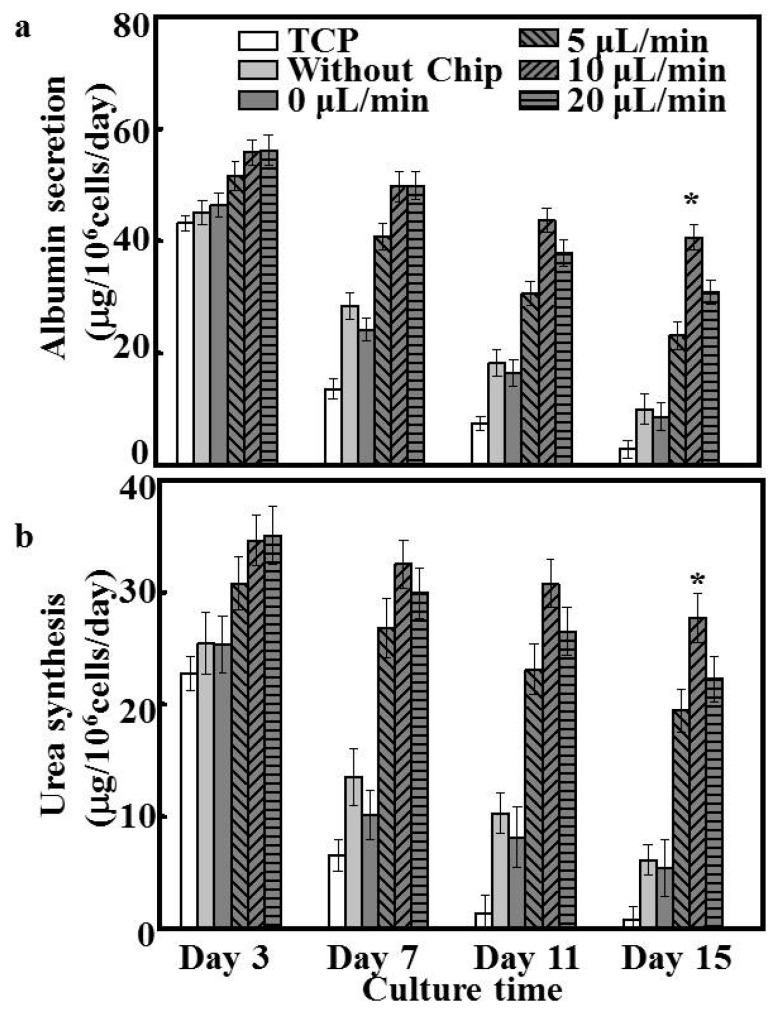
Liver-specific functions of hepatocytes on different substrates during 15 days of culture, including: (**a**) albumin secretion and (**b**) urea synthesis (*n* = 5); * *p* < 0.05.

The hepatocyte spheroid is considered to sustain cell viability for extended culture periods and maintain high levels of liver-specific functions [23]. Figure 5 summarizes the albumin secretion and urea synthesis of hepatocytes on different substrates. Hepatocytes cultured on TCP and patterned fibers rapidly lost their functional characteristics, and the level of albumin and urea production decreased throughout 15 days of culture. In addition, no significant differences were observed between patterned fibers without chip and the flow rate of 0 μL/min. Compared with patterned fibers without chip, the albumin and urea secretion of cells under the flow rate of 0 μL/min showed around 65% and 45% decrease after 15 days, indicating that patterned fibers do not represent a determining factor affecting the maintenance of liver function in the patterned fibrous mat-based microfluidic chip system. Although the albumin production of hepatocytes under dynamic conditions showed a slight decrease throughout 15 days of incubation compared to that of static culture (*p* < 0.05) (Figure 5a), hepatocytes under the flow rate of 5 μL/min sustained a significantly higher level at 23.14 ± 2.55 μg/10^6^ cells/day. As shown Figure 5b, similar results were noted for urea synthesis, for which hepatocytes under the flow rate of 5 μL/min also showed a slight decrease to about 19.47 ± 1.92 μg/10^6^ cells/day after 15 days of incubation. Of the three flow rates, hepatocytes cultured at the flow rate of 10 μL/min exhibited the greatest degree of albumin secretion and urea synthesis throughout the culture period, which was about 10- and 15-fold higher, respectively, than that of hepatocytes cultured on TCP for 15 days (*p* < 0.05). However, the albumin and urea production of primary hepatocytes perfused at 20 μL/min was lower than that of hepatocytes cultured at the flow rate of 10 μL/min, similar to the trend of viability and spheroid formation of the cultured hepatocytes.

### 3.6. Bile Canaliculi Formation by Hepatocytes

**Figure 6:**
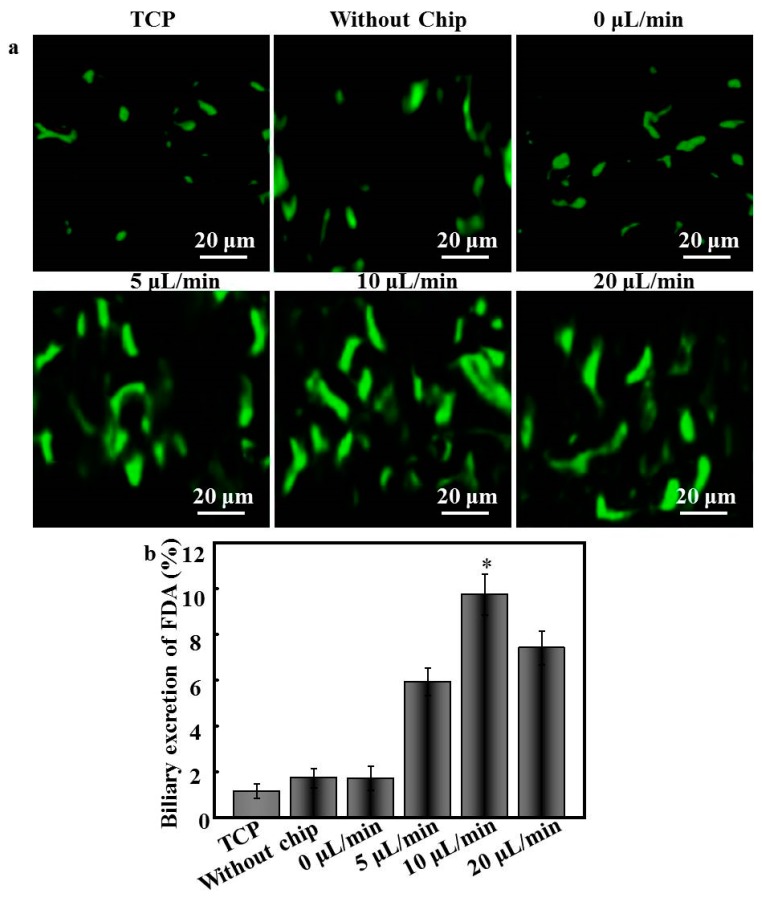
(**a**) CLSM images of fluorescein diacetate (FDA)-stained hepatocytes cultured on different substrates for 15 days; (**b**) quantification of fluorescence intensity of FDA-containing cells cultured for 15 days (*n* = 5); * *p* < 0.05.

Hepatocyte membrane polarity is evidenced by extended 3D bile canalicular structures, which are responsible for transport functions and drug metabolism [24]. In our study, the biliary excretory function of hepatocyte spheroids was investigated by incubation with FDA, which enters cells via passive diffusion and is hydrolyzed by intracellular esterases into fluorescein prior to excretion by bile canaliculi. Hepatocytes cultured under static systems exhibited subtle unconnected dots in the apical domain between adjacent hepatocytes, illustrating limited bile canaliculi structure formation (Figure 6a). Conversely, strong fluorescence signals were detected along the border of hepatocytes in all dynamic systems, indicating that those hepatocytes maintained their ability to uptake chemicals and efflux bile acid [25]. In particular, in hepatocytes cultured under the flow rate of 10 μL/min, the fluorescein appeared at the intercellular borders of hepatocytes and formed a tightly fused 3D morphology. Fluorescence quantitation, as shown in Figure 6b, further validated the observed differences, indicating that the dynamic conditions significantly enhanced the formation of bile canaliculi (*p* < 0.05), and that the flow rate of 10 μL/min exhibited significantly stronger biliary excretory function than the other rates (*p* < 0.05).

### 3.7. Toxicity Testing

**Figure 7:**
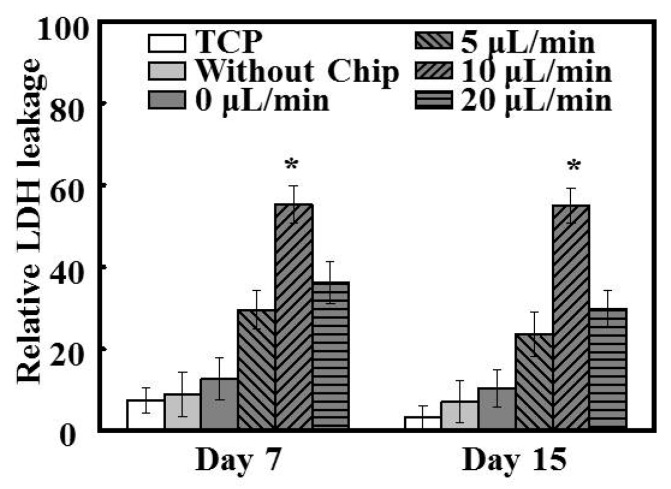
Relative lactate dehydrogenase (LDH) leakage from cells after 120 µg/mL silver nanoparticles (Ag NPs) treatment for 24 h at days 7 and 15 of culture, compared with the values without Ag NPs treatment (*n* = 5); * *p* < 0.05.

The toxic effects of Ag NPs were evaluated quantitatively utilizing the LDH assay, which detects the amount of LDH that leaks out through the plasma membrane of damaged cells. Figure 7 summarizes the relative LDH leakage from cultured hepatocytes on days 7 and 15 after treatment with 120 μg/mL Ag NPs for 24 h. Notably, a constant decrease in the relative LDH leakage was detected for hepatocytes under static conditions after Ag NP treatment. This indicated that under static conditions, the hepatocytes could not form spheroids and thus could not maintain their functions over a long period, resulting in a relative insensitivity to nanotoxicity. In contrast, the ability of hepatocytes under dynamic conditions to form compact spheroids led to their viabilities being significantly compromised compared to those of cells grown on TCP and patterned fibers throughout the study (*p* < 0.05). Furthermore, the consistency of the nanotoxicity response of hepatocytes under the flow rate of 10 μL/min on day 7 and day 15 was also notable; specifically, no apparent change in the relative LDH leakage from hepatocytes under this flow rate was observed following Ag NP treatment on days 7 (55.62% ± 4.53%) and 15 (54.48% ± 4.41%). This phenomenon indicated that hepatocytes under this flow rate condition were more sensitive to the Ag NP-induced toxicity than were cells under other growth conditions.

## 4. Discussion

Most previous bioreactor and microfluidic chips have been established to form hepatocyte spheroids by providing the beneficial effects of nutrients and oxygen as well as shear stress [26]. However, these involved 2D monolayer hepatocyte cultures. In the current study, we aimed to develop a 3D fluid cultivation system for long-term hepatocyte culture. In the native environment, hepatocytes are arranged into a single cordlike structure and are separated by sparse ECM [27]. To establish cell–cell and cell–ECM communication similar to that in native liver structures, scalable and readily available patterned fibrous mats and microfluidic chips were used in the 3D system.

In the current microfluidic system, the thickness of the patterned fibers was about 100 μm, which was lower than that of the probing channel and did not affect the microstructures of the fibers. The process of assembly essentially comprised liquid exchange and rendered the patterned fiber-embedded microfluidic chips highly compact. The morphology of patterned fibers was also considered critical for hepatocytes, as preliminary studies demonstrated that the crossing-points between fibers and fiber alignment might be important for increasing the initial attachment and spreading of hepatocytes. After patterned fibers were deposited on the collector, the adhesive forces remained between the patterned fibers and the collector such that it was not necessary to peel off the patterned fibers from the glass. Instead, the PDMS and the glass could be adhered together using a piece of Scotch Tape, which tightly adhered to the PDMS slab by van der Waals forces; notably, this adherence was crucial to the performance of the system. Unlike the other methods for chip assembly, our technique has several advantages: simple and inexpensive fabrication, no leakage, reversible sealing, and easy manipulation. Furthermore, the direction of patterned fibers aligned with the direction of flow, thus forcing the cells to migrate uniaxially, thereby concentrating the cells in close proximity to one another and likely accelerating aggregate formation. Accordingly, the hepatocyte aggregates on patterned fibers exhibited extensive cell–cell contact and tight junctions, and preserved their function and cell activity better than cells exposed to TCP over a 15-day culture period. Therefore, the inclusion of both channels and fibers allows this system to offer a large potential for use in miniaturized tissue engineering and high-throughput drug screening.

In addition, although continuous supply of fresh medium was provided to hepatocytes during the culture time, we noted that more dead cells occurred on TCP and patterned fibers without chip cultures under static systems, owing to limited diffusion. This finding was consistent with previous reports that microfluidic chips could provide a hydrodynamic environment and induce shear stress, and that nutrition could infiltrate into hepatocytes to significantly promote cell adhesion, and, accordingly, to better maintain the cell phenotype [28]. One notable aspect of our system was that few dead cells were observed under the flow rate of 10 μL/min, which might contribute to the balanced fluid movement of oxygen and nutrient exchange [29]. Conversely, the total dead cell numbers of hepatocytes cultured at flow rates of 5 and 20 μL/min increased. This phenomenon might be due to the extremely slow and fast flow, respectively, with the former offering insufficient supply of nutrients and oxygen to the cells and the latter subjecting hepatocytes to high laminar shear stress [30]. Generally, it is considered that the flow rate should be equal to the critical perfusion rate to guarantee that cells receive sufficient nutrients and oxygen in dynamic culture systems [31]. 

Notably, previous studies have shown that the size of the aggregates is crucial to hepatocyte function [32]. We further found that different levels of flow influenced the size distribution of the hepatocytes. During the first 72 h after seeding, small aggregates (40–50 μm) were formed; these clusters grew in size during the following 2 days, with the size of hepatocytes cultured under flow conditions gradually increasing as result of aggregation through cell–cell interactions and fluidic features. Hepatocyte spheroids under flow conditions became larger than those of under static conditions and visibly increased in size between day 7 and day 15, whereas the size of hepatocytes under static conditions remained the same during culture time, as was confirmed by immunostaining (Figure 4a). This result demonstrated the effect of continuous nutrient, oxygen, and cytokine transport and removal of metabolic wastes caused by interstitial flow [33]. Figure 4b shows that hepatocyte spheroids at flow rates of 5 and 20 μL/min increased gradually from day 1 (34.52 ± 2.48 μm and 42.78 ± 2.27 μm) to day 11 (61.06 ± 2. 82 μm and 75.47 ± 3.04 μm), whereas for the next 4 days they increased only marginally (71.08 ± 3. 02 μm and 76.86 ± 3.15 μm), possibly because hepatocytes within these flow rates were also subject to any significant mass-transfer limitations [34]. Although previous studies have shown that the central hepatocytes become hypoxic when the aggregate diameter is 100 μm or more [35], in the current study, hepatocytes cultured at the optimal flow rate of 10 μL/min formed spheroids with a maximum size of 105.33 ± 4.54 μm. 

To the best of our knowledge, perfusion systems further enhance hepatocyte function under dynamic culture conditions compared to that observed under static culture [36]. In the current study, patterned fibers and microfluidic chips were able to recapitulate cellular microenvironments in vitro on a microscale. Patterned fibers in the perfusion system provided ample adhesive sites and secure shields for hepatocytes to endure shear stress, and the cell arrangement could be easily manipulated and formed spheroids. Accordingly, the albumin and urea secretion for all dynamic culture groups exhibited higher yields than those obtained during static culture (Figure 5). Such marked maintainability is likely explained by the higher cell vitality and number of hepatocyte spheroids arising under dynamic culture. Bile canaliculi formation represents one of the key phenotypes of hepatocyte polarization, ensuring the efficient polarized transport of hepatic tissues. Compared to hepatocytes cultured under the static system, functional bile canaliculi networks were obviously present in the hepatocyte spheroids formed under dynamic culture [37]. Especially under the flow rate of 10 μL/min, bile canaliculi were well-developed, and the respective hepatocytes formed multiple junctions within spheroids. The ultrastructures of such bile canaliculi have been shown to facilitate the diffusive delivery of culture media to the center of hepatic spheroids, which is an important parameter affecting cell morphology, function, and physiological responsiveness [38]. Therefore, the patterned fibrous mat-based microfluidic chip likely represents a useful tool for fundamental studies of hepatic functions in physiological conditions and in more complex drug-transport studies.

Based on previously studies, exposure to 120 μg/mL Ag NPs was used to model nanoparticle toxicity in order to test the sensitivity, consistency, and feasibility of the system [39]. Owing to the well-maintained metabolic functions of hepatic spheroids, cells under dynamic culture exhibited higher sensitivity to Ag NP-induced toxicity than those grown under static conditions on both day 7 and day 15 (Figure 7). It has been extensively reported that sphere-shaped hepatic clusters are not only able to mimic the original morphology of the liver, but also provide functional hepatocytes in vitro [40]. Notably, the hepatocytes grown in the platform utilized in the current study could form physiologically relevant spheroids, potentially allowing the extension of the current level of cytotoxicity assessment from the cellular to the tissue level thereby reducing prediction error and improving prediction accuracy. In addition, following the treatment of hepatocytes with Ag NPs for 24 h, approximately 50% of the cells grown under perfusion culture had died. This phenomenon was similar to previous findings such as those by Firouz et al., who reported an Ag NPs IC_50_ value (the nanoparticle concentration causing 50% cell death) of 121.7 µg/mL [20]. Furthermore, the viability of hepatocytes under the flow rate of 10 μL/min after Ag NP treatment on day 7 and day 15 was similar, indicating that this flow rate achieved consistent, reliable, and high predictive values, and that it also extended the functional period up to 15 days. In contrast, hepatocytes under other growth conditions exhibited highly variable cell viability results at these time points following Ag NP treatment. Overall, however, hepatocytes that exhibited lower functioning on day 15 appeared to be more vulnerable to Ag NP-induced toxicity than those with poor function at 7 days (Figure 7), likely because of the absence of key physiological processes such as the transport of NPs via homotypic cell–cell interactions [41]. These results demonstrate the feasibility of hepatocytes cultured in this system as a potential in vitro testing model for the prediction of nanotoxicity, although further cellular and molecular investigations would be needed for a better understanding of the underlying mechanisms.

## 5. Conclusions

In this study, a new method to integrate patterned electrospun fibers within a microfluidic chip was developed and successfully demonstrated. The combined effects of microfluidic channels and patterned fibers on hepatocyte behavior were studied. Hepatocytes cultured in this system under an optimized flow condition exhibited restored hepatocyte polarity and biliary excretion, and maintained liver-specific functions over at least 15 days. Furthermore, hepatocytes under the flow rate of 10 μL/min produced sensitive and consistent Ag NP toxicity responses at different time points, demonstrating the feasibility of the patterned fiber-embedded microfluidic chips as a potential in vitro screening model to evaluate the potential toxicity of nanoparticles.
